# Supplementary material for: Patient-provider experiences with chronic non-communicable disease care during COVID-19 lockdowns in rural Uganda: A qualitative analysis
Source: PLoS One. 2023 Dec 14;18(12):e0295596. doi: 10.1371/journal.pone.0295596 (PMC10721044; doi:10.1371/journal.pone.0295596)
Supplement: S1 File — The interview guides used for participant interviews. (PDF) [file pone.0295596.s002.pdf]

Supplemental appendix 1a: Semi-structured qualitative patient interview guide

- Tell me about how you are doing these days.
  - Tell me about how your health is these days.

- Tell me about what a typical day was like for you during the time of lockdown (that is when the government asked everyone to stay at home in order to prevent the spread of corona virus).
  - In what ways was it different from a typical day outside that lockdown ?

- Tell me about what foods you were eating during those days of lockdown while staying home?
  - How was that compared to what you ate before that period of staying home?
  - How did the food you ate change during that time? (*Probe for food types/amounts eaten in one sitting/number of meals a day*)
  - Do you eat certain foods because of your medical condition? Did that change while enforced staying home?
  - Do you currently or previously take any alcoholic drinks? If so, I know the bars were closed during that time but you could buy alcohol from shops. However, did your intake of alcohol change during those days of staying home? (*Probe type/amount per sitting/number of times per day/week/month*)

  - Do you currently or previously use tobacco in any form for example smoking cigarette/pipe/stick or chewing ? If so, did your use of tobacco change during those days of staying home? (*Probe type/amount per sitting/number of times per day/week/month*)

- Tell me about going to the health center, clinic, or hospital during those days of staying home?
  - Did you have any need to see a healthy provider during that period of staying at home? If so, did you go to the health center, clinic, or hospital? Was it the facility you usually go?
  - If not, what made you select that particular facility? what difficulties did you have that made you not to go to the usual facility? How different was the facility you went to different for you? (Probe about distance, availability and type of clinical cadre, medication availability)

- What other challenges, not already mentioned, did you face during the days of staying at home?
  - How were these related to your health condition?
  - How was your overall health affected by those days of staying at home?

- How do you feel your care could have been improved by the government and health providers during the period of staying at home?
    - What would have made it easier to go to clinic? Or get medications?
    - To stay adherent to recommendations from health care providers (diet, smoking, and alcohol use)?

- Tell me about how you think telephones could be used in your medical care?
    - What information or services would you like to get over the phone?
    - What would you do think if a health care provider from the facility you go to called you about your health?
    - Who would you feel comfortable calling you on the phone about your health?

Supplemental appendix 1b: Semi-structured qualitative provider interview guide

- Tell me about your position at the clinic?
  - What does your day typically look like?
  - What are your responsibilities?

- How did your work change during the COVID pandemic?
  - How did your daily activities change?
  - How did your responsibility change, if at all?

- How did the clinic volume change during the pandemic?
  - Did you notice a change in the number of patients coming in?
  - (Probe approximate number of people, demographics characteristics like age group, sex, social class etc)

- How did the COVID-19 pandemic affect your NCD patients?
  - Did they come to clinic?
  - Were they able to follow life-style changes?
  - Were they able to get medications?
  - Who were the ones that you saw in clinic?

- What do you feel would improve care for patients with non-communicable diseases?
  - What around medications?
  - What around life-style modifications?
  - What around adherence?
  - What around technology?

- What would allow your clinic the capability to manage the full spectrum of NCD care?
  - Can you think of ways that would help you improve availability of medications?

- Are there mobile tools that would help improve care?
  - How would you imagine telephones helping NCD care?
  - For health care providers and the patients?

- How do you think technology could be helpful in your clinic activities?
  - Would you imagine using it during appointments? Or after?
  - Who would you be communicating with?
