# Supplementary material for: Patient-provider experiences with chronic non-communicable disease care during COVID-19 lockdowns in rural Uganda: A qualitative analysis
Source: PLoS One. 2023 Dec 14;18(12):e0295596. doi: 10.1371/journal.pone.0295596 (PMC10721044; doi:10.1371/journal.pone.0295596)
Supplement: S2 File — The Master Codebook used to analyze the study’s data. (PDF) [file pone.0295596.s003.pdf]

**NCD experiences during COVID-19 lockdowns**
**Master Codebook**
**Version 1.0, November 12, 2023**

**Introduction to Codebook:**
This codebook is organized into four sections: (1) Question-driven Codes for non-communicable disease (NCD) care, (2) Question-driven Codes for COVID-19 lockdowns, (3) Question-driven Codes for improvements to care, and (4) Open Codes (NCD care, COVID-19 lockdowns, improvements to care). The Question-driven Codes were derived directly from participants' answers to interview questions and reflect key domains from the interview guides. The Open Codes were developed inductively from recurring phrases or concepts in the interviews that were judged to be particularly significant.

**Definition of Key Terms:**
Code labels: short phrases appearing in bold at the beginning of each code description (e.g. "Medication Adherence").
Code definitions: statements elaborating the meaning of each code label.
Participant: study participants
NCD: non-communicable disease
PA: Patient participant
PR: Provider participant

**Directions to Coders**:
Coders will review the code labels, code definitions and illustrative examples in this document. Coders will then identify sections of interview transcript text that correspond to the code definitions and assign the appropriate code label or labels to those sections. When a section of text corresponds to more than one coding category, it will be "double" or even "triple"-coded such that it is assigned to each category to which it corresponds.

**I. Question-driven Codes for NCD care**

**Medication Adherence**
*Definition:* participant descriptions of medication adherence. These may include statements about (1) timing of administration, (2) method of administration, (3) where medications are purchased, and (4) pill burden.

> *Examples:*
> *"When I wake up I first swallow my hypertension medicine after taking my breakfast and then inject myself with insulin at around 8:30 am with 15 cc and in the evening 10 cc" PA 10*
>
> *"Hypertension drugs are so expensive, most of the time I do not buy enough dosage because I do lack money. Even the little I get access to gets finished so fast, I am forced to do without drugs for some time. By the time I come back to the hypertension clinic on my clinic day, the health workers find it [blood pressure] has already shot up." PA 5*

*"We are supposed to be giving our patients medicines for one month but because we do not have enough, we are forced to give them only for a half a month because we do want other patients also to get something. So what we do we give them fifteen tablets and tell them to go and buy the rest from outside. Then the other fifteen we give them to another patient also to survive on." PR 6*

*"A lot of people out there suffer from pill burden, and they are tired of taking the pills every day. You get a patient with multiple diseases like HIV, hypertension, and diabetes, who takes medicine for all those conditions at the same time. It becomes too much because these medicines have to be taken for the rest of their lives." PR 6*

**Social stressors**
*Definition:* participant descriptions of social stressors and stigma that impact NCD care, both for the patients and the providers.

*Examples:*
*"I get stressed. I have several children with their challenges and other family issues." PA 1*

*"I was stigmatised [for my condition]. I hated myself, and at one point I wished I had died because it reached an extent that I became a problem to other people." PA 20*

**Financial challenges**
*Definition:* participant descriptions of financial challenges that impact their NCD care.

*Examples:*
*For hypertension [drugs], I do not usually have them. They are so expensive, and I am unable to access them because of not having money." PA 6.*

*Most of our colleagues die because of the stress of looking for money and others because they cannot afford to buy a single tablet which is more than a thousand shillings. You have to spend this money throughout your life. Where can a common person like me get such money." PA 5*

**Life-style changes**
*Definition:* participant descriptions of life-style changes they have made due to their NCD diagnosis, often at the recommendation of a clinician. These include (1) exercise, (2) dietary changes, and (3) alcohol and cigarette use.

*Examples*:
*"I used to engage in a lot of physical activities, I used to work so hard but later I stopped all that because of my hypertension condition. I lost energy, I became too weak, I could*

*no long walk long distances, not at all. My work was basically to keep at home seated and being spoon fed like a baby." PA 2*

*I eat the usual local food, but my eating habits are not good. When [the clinicians] advise you to feed on certain foods, my wife ends up not being able to do it." PA 15*

*"I took beer for a long time, I really took a lot, I could not limit myself. It was too mich. So when I went for medical treatment I was told to stop drinking and that is how I stopped." PA 6*

**II. Question-driven Codes for COVID-19 lockdowns**

**Food insecurity**
*Definition:* participant descriptions of how access to food changed during COVID-19 lockdowns. This included (1) increased price of food, (2) fewer food options, and (3) less food available at home.

*Examples:*
*"We used to get hungry and there was nothing to do, only to stare at each other and wait for whatever comes on our way. When children asked us for what to cook, we used to pretend as if we have not heard anything and provide whatever we could. If we managed to get some food, it would not enough." PA 8*
*"There was no food supply, people were not working, no money and people were basically yearning for whatever they could just throw in their stomachs." PR 1*

*"There was no money during the COVID-19 lockdown. We did not have a variety of food and so we used to just eat whatever we came across." PA 5*

**Financial changes**
*Definition:* participant descriptions of changes to their finances that occurred during COVID-19 lockdowns. This included (1) loss of income or employment, and (2) increased cost of basic goods and services.

*Examples:*
*"COVID-19 really made my life miserable. I did not know what was going to happen next; no money, no movements, no medicine. I was too weak. I was not myself and I was always dizzy. I can tell you l almost lost hope. PA 19*

*"There was no money during the COVID-19 lockdown. We did not have a variety of food and so we used to just eat whatever we came across." PA 5*

**Transport changes**

*Definition:* participant descriptions of changes in transport that occurred during the COVID-19 lockdowns. This included (1) lack of availability of transportation, (2) different modes of transportation available, and (3) difficulties in getting patients to higher level health facilities.

> *Examples:*
> "*Remember most of us we are from poor societies with no money. In fact, after this interview, I am going to use my feet and walk home, which is about 10 kilometres from here." PA 8*
>
> *"Transport was restricted and most of the people who were able to come to the facility were those that came from nearer and those who were caught up by the lock down near this facility." PR 1*

**Facility-level changes**
*Definition:* participant descriptions of how NCD care at health facilities changed during the COVID-19 lockdowns. This included (1) deprioritizatoin of NCD care, (2) medication shortages, (3) changes to provider workflow, and (4) changes to patient population.

> *Examples:*
> *"All the attention at the hospital was shifted from hypertension and other illnesses to COVID-19 strictly." PA 8*
>
> *"The Ministry of Health would take a very long time to bring medications to the facility and that impacted us tremendously. I think there were a lot of bureaucracies securing medicine from national medical stores and that caused a lot of delay in delivering medicine at the facility. They usually send us medicines quarterly but during COVID, deliveries often took more than 6 months to come. When patients come and don't find any medicines, they get frustrated." PR 11*
>
> *"Transport was restricted and most of the people who were able to come to the facility were those that came from nearer and those who were caught up by the lock down near this facility." PR 1*

**Alternative forms of care**
*Definition:* participant descriptions of alternative forms of health care sought during the COVID-19 pandemic. This included (1) alternative public or private facilities, and (2) use of local herbs for treatment.

> *Examples:*
> *"It was not comfortable getting treatment from those [private] clinic because they never used to test or examine us, instead they would just give us drugs and that was all." PA 9*
>
> *"I had to look for alternative means so that I can stay alive and that was the local herbs, I used those local herbs through the whole COVID-19 lockdown until things got better and I was able to access hypertension drugs again." PA 8*

*"For the hypertension patients, everyone is for him or herself, those who could manage, would come and others would buy medicine from the pharmacies near them. Then those who did not have money had to give up and use local herbs. Most of them missed doctor's consultations, health education and physical examination, they missed all that." PR 5*

**III. Question-driven Codes for improvements to care**

**mHealth**
*Definition:* participant views on how mHealth could impact NCD care. This included (1) perceptions of mHealth, (2) participant suggestions for specific types of intervetnions, and (3) challenges with implementing mHealth.

*Examples:*
*"I think the use of technology in the provision on health services to patient with non-communicable diseases is a good idea and it will help in reducing the workload and reduce on the burden that are faced by patients. Technology also will help us both the client and the health workers do the right thing and improve on service delivery." PR 6*

*"[Health workers] can also use telephone contacts to reach out to patients, ask them how they are feeling and get feedback as soon as possible. They can also advise patients, give health education, and provide reminders by phone in case movements are restricted." PA 5*

*"Almost every person these days owns a cell phone, and these phones can help in passing on information, health education counselling, and reminders to people out there very cheaply and faster." PR 3*

**Decentralized care**
*Definition:* participant descriptions of how NCD care could be brought more locally. This included (1) clinic availability, (2) clinic structuring, (3) improved medication availability, and (4) improved diagnostic availability.

*Examples:*
*"Hypertension drugs should be brought to health centres II and III so that those who cannot afford going to the main hospital also have a chance of accessing care without spending a lot on transportation. That will save [patients] money, time, and effort to travel such long distances." PA 3*

*"The government should have done a lot to help us, provide us with free medicines and bring them closer to health centres that are accessible and easy to reach" PA 2*

**Community outreach/community health workers**
*Definition:* participant description of

This included (1) use of community health workers, (2) increased community outreach for sensitization, treatment, and diagnosis, and (3) patient support groups.

*Examples:*
*"There are some implementing partners [in the community] who facilitate care like TASO and others. Most that is done with HIV patients, but I believe if the same can be done with people living with non-communicable diseases, it would really help a great deal." PR 2*

*"The government should have been able to train and support health workers to do more community outreaches, and get us our medical treatment in our villages so that we do not suffer and be left alone to die in our villages." PA 18*

*"When the patients join the support group, the first step is to make a contribution. If there are no drugs for them here at the clinic, the association procures their own medicine and then gives them to patients." PR 4*

**Training**
*Definition:* participant descriptions of how to improve training of health providers on the care of NCDs.

*Examples:*
*"I think health workers needs to have more refresher trainings on how best they are supposed to manage NCD patients. Most of the health workers are working on the trainings that they got from school a long time ago." PR 2*

*"Continuous training in NCD care is needed, but also the number of staff in our unit should be scaled up in order to reduce on the workload within our clinic." PR 4*

*If there were many counsellors, they would be able to help out and counsel these patients so that they modify their life style and free themselves with non-communicable diseases." PR 5*

## IV. Open Codes

**HIV treatment comparisons**
*Definition:* participant statements that compared NCD care to HIV care. This included (1) comparisons between ease of access for services, (2) examples of HIV interventions that could be applied to NCD care.

*Examples:*
*"People living with HIV, on the other hand, are given free drugs. But us who just get these diseases without our knowledge, there is totally no effort at all to help us." PA 5*

*"Patients living non-communicable diseases need constant and serious follow up just like other disease like HIV. We could try to engage them in community follow-up, sensitise them, and teach them the best way of taking their medicine very well." PR 4*

*"There are some implementing partners who facilities like TASO and others. Most that is done with HIV patients, but I believe if the same can be done with people living with non-communicable diseases, it would really help a great deal." PR 2*

**Changed health during COVID-19 lockdowns**
*Definition:* participant descriptions of physical and mental health changes that occurred during the COVID-19 lockdowns.

*Examples:*
*"COVID-19 really made my life miserable. I did not know what was going to happen next; no money, no movements, no medicine. I was too weak. I was not myself and I was always dizzy. I can tell you l almost lost hope. PA 19*

*"It was a total mess, most of our colleagues lost their lives because they could not access their drugs and there was no one to think about them, everyone was just for him or herself, only the strongest survived. Some of us just survived by the mercies of God. We practically lived in constant fear, hopeless lives and with a lot of worries in every aspect of life." PA 16*

*"Patients living with non-communicable diseases would not come to the hospital to seek treatment, they got so sick. Some of them ended up dying in their homes with no help in the villages and communities." PR 4*

**Fears of acquiring COVID-19**
*Definition:* participant descriptions that related to fears of contracting COVID-19. These included (1) avoidance of public places, (2) avoidance of social interactions, and (3) avoidance of health facilities.

*Examples:*
*"There was also an element of stigma. Patients used to fear coming to the facility because they thought they would catch COVID-19." PR 5*

*"We started fearing fellow human beings. As health workers, we would limit physical contact with patients in the health facility, and some of the vital measurements like the blood pressure, height, weight, and others were not done unless the patient was in a critical condition." PR 8*

*"I used to fear walking beyond my compound to the trading centre because of fear of contracting COVID-19. We were advised to keep away from people and always keep a social distance." PA 1*
