# Supplementary material for: Patient-provider experiences with chronic non-communicable disease care during COVID-19 lockdowns in rural Uganda: A qualitative analysis
Source: PLoS One. 2023 Dec 14;18(12):e0295596. doi: 10.1371/journal.pone.0295596 (PMC10721044; doi:10.1371/journal.pone.0295596)
Supplement: Attachment — Submitted filename: PEER REVIEW OF MANUSCRIPT FOR PLOS ONE.docx [file pone.0295596.s004.docx]

PEER REVIEW OF MANUSCRIPT FOR PLOS ONE

TITLE: Patient-provider experiences with chronic non-communicable disease care during1

COVID-19 lockdowns in rural Uganda

ABSTRACT:

The abstract is concise and well written

INTRODUCTION

The introduction is very short, resulting an inadequate justification for the study. Many of the references cited in the discussion, could be brought into the introduction, as this was known already, including the impacts of lockdowns on NCDs in other countries, and the possibilities of mHealth. A funnel of existing evidence, starting with a worldwide perspective, and narrowing to LMICs and Africa, then to Uganda, would be helpful to set the scene. It is clear from the interview guide that the discussions about mHealth were the intention from the start, so bringing that literature into the introduction would be more appropriate.

METHODS

The choice of patients attending Mbarara Regional Referral Hospital and HC IV needs greater justification, as these are likely to be a specific group of patients requiring a higher level of care than those with uncomplicated NCDs who might be managed at a lower level of care. Was this a convenience sample, or was this a deliberate choice?

The selection of healthcare provider participants who were “purposively recruited in conjunction with the head of each health facility” needs further elaboration, in terms of the criteria for inclusion or exclusion beyond those stated in lines 95 to 98, as this could have unintentionally introduced a particular reflexivity bias. How a medical records officer and a laboratory technician based in a hospital, for example, would have particularly deep insight into NCD care during the lockdowns, is difficult to understand, compared to a community health worker for example.

The role and positions of the authors conducting a qualitative study, needs to be stated in the methods section, in terms of their positionality in relation to the participants and how this was mitigated, in terms of reflexivity. Then in the discussion section, the ways in which the authors who “have worked as physicians in rural areas across SSA” might have influenced the results, deserves a mention alongside any other limitations of the study.

RESULTS

These are well presented, with sufficient verbatim quotes to back up each statement.

DISCUSSION

The discussion is solid but not ground-breaking. What might make the whole manuscript more impactful would be to shift some of the references to the introduction, allowing space for a more critical discussion in relation to more recent literature in the topic. This should be followed, as mentioned above, by a reflection on the limitations of the study, as well as directions for future research. This goes beyond preparing for future lockdowns, to the implications arising from the study for routine NCD care outside of exceptional circumstances such as a pandemic.

REFERENCES

These need a lot more attention to detail in terms of completeness and formatting. There are too many shortcomings to mention each in detail, and this section should be checked for accuracy after revisions.

TYPO’S

Line 35: pre-existing

Line 110: NCD care

Line 309: “pain points” is a particular phrase that requires modification for an international audience

Line 398: strengthen (not strengthening)
