# Supplementary material for: Patient-provider experiences with chronic non-communicable disease care during COVID-19 lockdowns in rural Uganda: A qualitative analysis
Source: PLoS One. 2023 Dec 14;18(12):e0295596. doi: 10.1371/journal.pone.0295596 (PMC10721044; doi:10.1371/journal.pone.0295596)
Supplement: Attachment — Submitted filename: Editors comments_November_2023.docx [file pone.0295596.s005.docx]

**Editor’s comments**

Title: Kindly identify the topic as: A qualitative study

**Abstract**

The abstract is well written

**Introduction**

The introduction is too short. Add a research question/objective at the end of the introduction. Kindly follow Review1 comment on introduction

**Methodology**

1. Separate recruited participants: a. Patients and b. Providers: kindly explain in details how each group of participants was recruited. For provider participants, it seems you have used a snowballing method where a manager refers the interviewer to a specific provider participant among his/her workers. Kindly make it clear. For patient participants, it seems this was purposeful sampling, kindly make it clear how the sampling process was conducted for each group in a separated paragraph

2.Kindly add a paragraph of Inclusion & exclusion criteria for both groups, a. patient participants and b. provider participants

3. For interviews, we understand some participants were interviewed in Local language (Runyankore and Rukiga) and English. Clarify how you translated and translated back the questionnaires/results from local language to English after interviews

4. There should be a strong justification why you included a lab technician and a medical record officer. For a midwife, unless she/he is a health center manager or deals with chronic diseases in pregnancy, yet it is clear that pregnant women were excluded. A strong justification is needed. For example, a laboratory technician can know when the reagents are available but rarely know if chronic disease medicines are available or not. (For example, if the laboratory technician participates in Health center management meetings, he/she can get the right information). Kindly make it clear

I don’t comeback to “reflexivity”, reviewer1 has commented on this.

**Findings**

I am having a big concern that all participants (both providers and patients) can be recognized easily by reading this paper. Anyone from the Mbarara hospital who reads this paper can recognize most of the participants. How? In the interview, you only have one clinical medical records officer and a clinical laboratory technician. These participants are known to have participated in the study and may be recognized easily.

Reporting both (gender and age) expose patients to be recognized by the hospital managers in a small sample size. These characteristics expose them to their identity.

To avoid the risk of exposing participants, kindly identify their quotes using a code. For example; for providers, allocate PR and add a number (PR1: provider1, PR2,…) and patients (PA1, PA2, PA3: patient1, patient2,…..). You can decide to code any how you want. Then keep the legend of your codes in your records or dataset

The following are the reported quotes. Make sure to change for all quotes with codes.

“ Line 169: (female patient, age 86) and Line 174 (Male patient, 70), Line 183: clinical nurse, Line 205(female patient, 53), Line 210-211 (male patient, 66), Line 221 (male patient, 70), Line 226 (female patient, 56), Line 228 (Clinic midwife), Line 244 (Female patient, 58), Line 254 (Clinic midwife), Line 257 (Female patient, 63), Line 261 (Clinic medical records officer), Line 269 (Female patient, 58), Line 272 (Clinic nurse), Line 293 (Female patient, 58), Line 299 (clinic nurse), Line 305-306 (male patient, 47), Line 325 (Female patient, 38), Line 332 (Female patient, 40), Line 344 (Male patient, 70),Line 349 (Clinic nurse), Line 357 (Clinic nurse), Line 367 (Clinic medical records officer), Line 377 (Clinic nurse)”

You are doing a good synthesis in between the findings but you are not supporting your arguments with citations.

Kindly support your arguments with good academic support (i.e : line 198-201: this strong arguments need to be supported by a citation). Some paragraphs need a synthesis with good arguments supported by citations instead of a simple reporting.

Line 214-217: This good argument needs a citation support

Line 233: Kindly use only COVID-19 pandemic: not epidemic

Line 280-Line 285; Is this same clinical nurse? Clinical nurses are many and they are only reported as “clinical nurse”, therefore confusing whether is the same nurse or not. Reason why the coding is required.

Line 308: The heading “5. Recommendations for NCD care improvement”. The numbering of this heading is confusing. It has the numbering like “Themes”. This is number 5 making impression that it is “theme5” yet it is not. Kindly check and remove the confusion on numbering

Line 317-320: Kindly support these arguments with a citation

**Discussion & Conclusions:** The discussion & conclusions are well done

Kindly follow PLOS ONE formatting and revise English grammar, typos and errors

<https://journals.plos.org/plosone/s/submission-guidelines>
